# Barriers, Opportunities, and Challenges in Addressing Disparities in Diet‐Related Cardiovascular Disease in the United States

**DOI:** 10.1161/JAHA.119.014433

**Published:** 2020-03-23

**Authors:** Penny M. Kris‐Etherton, Kristina S. Petersen, Gladys Velarde, Neal D. Barnard, Michael Miller, Emilio Ros, James H. O'Keefe, Kim Williams, Linda Van Horn, Muzi Na, Christina Shay, Paul Douglass, David L. Katz, Andrew M. Freeman

**Affiliations:** ^1^ Department of Nutritional Sciences The Pennsylvania State University University Park PA; ^2^ Division of Cardiology Department of Medicine University of Florida Jacksonville FL; ^3^ Adjunct Faculty George Washington University School of Medicine Physicians Committee for Responsible Medicine Washington DC; ^4^ Department of Medicine University of Maryland School of Medicine Baltimore MD; ^5^ Lipid Clinic Endocrinology and Nutrition Service Institut d'Investigacions Biomediques August Pi Sunyer Hospital Clinic University of Barcelona, and Centro de Investigación Biomédica en Red (CIBER) Fisiopatología de la Obesidad y Nutrición Instituto de Salud Carlos III Madrid Spain; ^6^ Saint Luke's Mid America Heart Institute School of Medicine University of Missouri–Kansas City MO; ^7^ Rush University Medical Center Chicago IL; ^8^ Department of Preventive Medicine Feinberg School of Medicine Northwestern University Chicago IL; ^9^ Center for Health Metrics and Evaluation American Heart Association American Heart Association Dallas TX; ^10^ Wellstar Medical Group, Metro Atlanta Cardiovascular Medicine Atlanta GA; ^11^ Yale‐Griffin Prevention Research Center Derby CT; ^12^ Division of Cardiology Department of Medicine National Jewish Health Denver CO

**Keywords:** cardiovascular disease prevention, cardiovascular disease risk factors, diet, disparities, nutrition, social determinants, Cardiovascular Disease, Diet and Nutrition, Race and Ethnicity, Risk Factors

## Abstract

In the United States, cardiovascular disease (CVD) is the leading cause of death and disability. Suboptimal diet quality is responsible for a greater percentage of CVD‐related morbidity and mortality than any other modifiable risk factor. Further troubling are the stark racial/ethnic and socioeconomic disparities in diet quality. This represents a major public health concern that urgently requires a coordinated effort to better characterize the barriers to healthy dietary practices in population groups disproportionally affected by CVD and poor diet quality to inform multifaceted approaches at the government (policy), community environment, sociocultural, and individual levels. This paper reviews the barriers, opportunities, and challenges involved in shifting population behaviors, especially in underserved populations, toward healthy dietary practices. It is imperative that public health policies address the social determinants of nutrition more intensively than previously in order to significantly decrease CVD on a population‐wide basis.

Cardiovascular disease (CVD) is the leading cause of morbidity and mortality in the United States.^1^ Despite significant progress in the past 40 years,^2^ reductions in the CVD mortality rate have slowed after 4 decades of decline; between 2010 and 2015, CVD deaths increased, although the age‐adjusted death rate declined by 1.8% between 2015 and 2016.^3^ Arnett et al^4^ attributed the deceleration in CVD mortality decline to increasing obesity prevalence, which is a direct result of suboptimal dietary habits. In the United States, approximately half of all CVD‐related disability and death is attributed to poor diet quality, making it the leading cause of CVD.^1^ Disparities in diet quality exist by race, ethnicity, and socioeconomic status (SES).^3^, ^5^, ^6^ Diet‐related disparities mirror the disproportionate burden of CVD in underserved populations.^3^ The purpose of this article is to summarize the disparities in diet quality that exist in the United States as it relates to CVD, and discuss barriers and strategies to improve overall diet quality with a focus on the social determinants of CVD and poor diet quality.

## Disparities in CVD and CVD Risk Factors

In the United States, the prevalence of CVD (coronary heart disease [CHD], heart failure, stroke, and hypertension) is 48.0%.^3^ However, some population groups experience a substantially greater burden of CVD and poorer outcomes.^7^ It is well established that CVD disproportionately affects non‐Hispanic (NH) blacks and individuals of low SES. However, these disparities are complex and multidimensional and often individuals have >1 socioenvironmental factor associated with greater CVD burden, and thus synergistic effects may exist.^8^ For example, in the United States, the prevalence of CVD is ≈7 to 10 percentage points higher in NH blacks compared with NH whites; CVD prevalence is comparable or lower than the national average in Hispanics and NH Asians. Similar disparities exist in the prevalence of stroke, which is higher in NH blacks compared with NH whites, Hispanics, and NH Asians.^3^ However, a strong inverse SES gradient exists such that NH blacks with lower SES have a greater burden of CVD than those of higher SES.^9^

Incidence of CHD is also greater in NH blacks versus NH whites. The most recent data from 2 large US cohorts show that the age‐adjusted incidence of fatal CHD events is ≈2‐ and 1.7‐fold higher in NH black men versus NH white men aged 45 to 64 and ≥65 years, respectively.^10^ In women, incidence of fatal and total CHD events was 1.44‐ to 2.61‐fold higher in NH blacks versus NH whites aged 45 to 64 years. NH black women aged ≥65 years had a higher incidence of fatal CHD than NH white women (hazard ratio, 1.57; 95% CI, 1.01–2.43). In these analyses, after further adjustment for the social determinants of health and major CVD risk factors, incidence of fatal, nonfatal, and total CHD was similar in NH blacks and NH whites.^10^ This underscores the contribution of social determinants to the burden of CVD, and although it is difficult to disentangle the relative contribution of individual determinants, significant opportunities exist to reduce the burden of CVD by addressing social determinants of health.^8^

**Figure 1:**
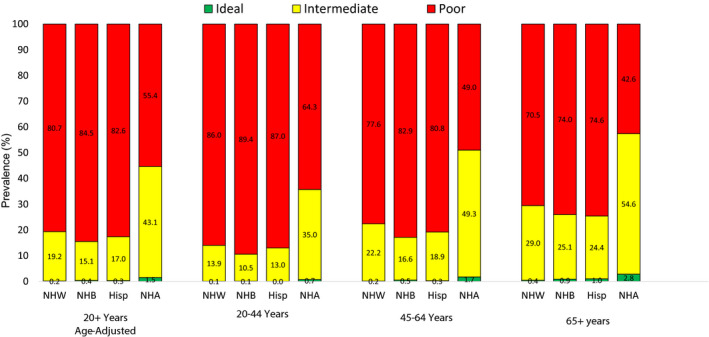
**Prevalence of poor, intermediate, and ideal scores for the dietary component of the American Heart Association's Ideal Cardiovascular Health Definition in US adults aged ≥20 years (age standardized) and selected age groups by race/ethnicity.** Source: National Center for Health Statistics, National Health and Nutrition Examination Survey, 2015 to 2016. Hisp indicates Hispanic; Ideal, 4 to 5 ideal components; Intermediate, 2 to 3 ideal components; NHA, non‐Hispanic Asian; NHB, non‐Hispanic black; NHW, non‐Hispanic white; and Poor, 0 to 1 ideal components.

Disparities also exist in the presence of CVD risk factors. The American Heart Association (AHA) defines ideal cardiovascular health by 7 core health behaviors (smoking, physical activity, diet, and body mass index) and health factors (cholesterol, blood pressure, and glucose control) (Table S1).^11^ Overall, the prevalence of ideal cardiovascular health is low (for all groups, <30%); however, it is much lower in NH blacks (10.6%) and Hispanics (14.2%) than it is for NH whites (19.4%) and NH Asians (29%).^3^ Similarly, low SES strata have a disproportionate burden of traditional CVD risk factors compared with those of higher SES.^12^, ^13^ Of particular concern is the prevalence of US adults with an ideal score for the dietary component (Healthy Diet Score; indicating a healthy diet), which is 0.2%.^3^ The vast majority of US adults receive a poor score (indicating a suboptimal diet); on average, 82% of individuals aged 20 to 49 years and 73% of individuals aged ≥50 years have a poor diet score. However, NH blacks and Hispanics tend to have greater prevalence of a poor diet score than NH whites and NH Asians (Figure 1).

## Disparities in Diet Quality

Disparities in diet quality by race/ethnicity, education level, income, and use of food assistance programs in the United States are well documented^14^, ^15^, ^16^ (Figures S1 through S4). Brown et al^16^ reported that for US adults aged >25 years without prevalent CVD, the number of NH blacks with a poor diet was greater than the number of NH whites by a magnitude of 6.8% to 11.7% percentage points from 1988 to 2010. However, no difference in the percentage of NH blacks and NH whites with poor diet quality was detected in 2011 to 2014, which was largely because of declining diet quality in NH whites. Rehm et al^15^ conducted similar analyses, but did not exclude individuals with CVD, and found that the percentage of NH white adults with a poor diet, defined by the AHA Healthy Diet Score, decreased significantly from 2003 to 2012 (53.9% to 42.8%). This contrasts with the findings of Brown et al,^16^ and may be explained by the inclusion of individuals with CVD in the analyses because dietary changes can occur in response to a CVD diagnosis.^17^ Rehm et al^15^ also reported no change in the proportion of NH black or Mexican American adults with poor diet quality during the period of study. Furthermore, the percentage of low‐income adults with poor diet quality was minimally changed or unchanged from 2003 to 2012, in comparison to a significant decline observed in the percentage of higher‐income adults classified as having a poor diet; these analyses were not adjusted for race/ethnicity or other social determinants of health. Similarly, Wang et al^14^ reported an increase in diet quality, defined by the Alternate Healthy Eating Index‐2010 (AHEI‐2010), in a representative US population sample, from 39.9 points in 1999 to 2000 to 46.8 points in 2009 to 2010 (linear trend *P*<0.001). In agreement with the findings of Rehm et al,^15^ disparities in the change in diet quality over time were observed; NH whites had an increase in diet quality from 1999 to 2010 (mean adjusted score, 34.1 points; 95% CI, 33.2–35.0 to 36.3 points; 95% CI, 35.6–37.0; *P*<0.001), whereas no changes in diet quality were observed for NH blacks (mean adjusted score, 33.5 points; 95% CI, 32.1–34.8 to 34.6 points; 95% CI, 33.5–35.6; *P*=0.06) or Mexican Americans (mean adjusted score, 35.7 points; 95% CI, 34.3–37.0 to 37.0 points; 95% CI, 36.8–37.2; *P*=0.10).^14^ Furthermore, individuals of high SES had higher diet quality at all time points over the decade of follow‐up compared with individuals of low and medium SES, after multivariate adjustment including education attainment and race/ethnicity; consistent improvements in diet quality over time were only observed with high SES. Kirkpatrick et al^18^ and Hiza et al^19^ reported similar findings; individuals in lower‐income households had poorer diets than their higher SES counterparts.

Diet disparities also exist by use of food assistance programs. Zhang et al^20^ reported no change in the proportion of Supplemental Nutrition Assistance Program (SNAP) participants and income‐eligible nonparticipants classified as having a poor, intermediate, or ideal AHA Healthy Diet Score from 2003 to 2014. In contrast, for higher‐income adults, the percentage of individuals with poor diet quality declined (−10.8 percentage points; 95% CI, −15.2 to −6.5 percentage points), and increases in the proportion with intermediate (9.6 percentage points; 95% CI, 5.3–13.9 percentage points) and ideal (1.2 percentage points; 95% CI, 0.3–2.2 percentage points) diet quality were observed. Similarly, an analysis of 4211 low‐income adults from the 2003 to 2010 National Health and Nutrition Examination Survey data set showed that SNAP participants had poorer overall diet quality, assessed by the HEI‐2010, and lower scores for fruits and vegetables, seafood and plant proteins, and empty calories compared with low‐income nonparticipants, but had comparable scores for whole grain, refined grain, total dairy, total protein, fatty acid, and sodium intakes.^21^ Likewise, a 2015 US Department of Agriculture (USDA) study comparing SNAP participants with income‐eligible nonparticipants showed that SNAP participants consumed more calories from solid fats, added sugars, soda, and alcohol, consumed fewer vegetables and fruits, and had poorer overall diet quality.

In the United States, disparities in diet quality exist by race, ethnicity, SES, education attainment, income, and use of food assistance programs. Although modest and clinically relevant improvements in diet quality have occurred over the past 2 decades at a population level, some racial and ethnic groups and those of low SES are disproportionally affected by poor diet. This is a key area requiring intervention because of the well‐established causal relationship between poor diet and increased risk of CVD.

A heart‐healthy diet is the cornerstone of atherosclerotic CVD prevention and treatment. Contemporary recommendations, from the AHA/American College of Cardiology, the USDA, and the Department of Health and Human Services, promote a healthy dietary pattern abundant in fruits, vegetables, whole grains, legumes, nuts, and seeds, that includes lean unprocessed protein sources (including poultry and seafood), fat‐free or low‐fat dairy products, and liquid nontropical oils.^4^, ^22^, ^23^, ^24^

Robust evidence demonstrates the cardiovascular benefits of healthy dietary patterns.^25^, ^26^, ^27^, ^28^, ^29^, ^30^, ^31^, ^32^, ^33^, ^34^ Observational studies consistently show a dose‐response inverse relationship between diet quality and CVD morbidity and mortality. Sotos‐Prieto et al^33^ reported an 8% to 17% reduction in total mortality and a 7% to 15% reduction in cardiovascular mortality per 20‐percentile increase in diet quality, over a 12‐year period, in the NHS (Nurses’ Health Study) and the HPFS (Health Professionals Follow‐Up Study). Thus, incremental improvements in diet quality can have meaningful cardiovascular benefits. These observational data are supported by the findings of the landmark PREDIMED (Prevención con dIeta Mediterránea) clinical trial, conducted in Spain, which showed that greater adherence to a Mediterranean diet reduced CVD events by ≈30% compared with a control group given recommendations to reduce intake of fat,^34^ although it is unclear how these results extrapolate to populations outside Spain. In PREDIMED, compared with the control group, the Mediterranean diet with extra virgin olive oil and the Mediterranean diet with mixed nuts lowered the CVD event rate from 11.2 per 1000 person years (95% CI, 9.2–13.5) to 8.1 per 1000 person years (95% CI, 6.6–9.9) and 8.0 per 1000 person years (95% CI, 6.4–9.9), respectively.

## Barriers to a Healthy Diet and Improving Diet Quality in Underserved Populations

**Figure 2:**
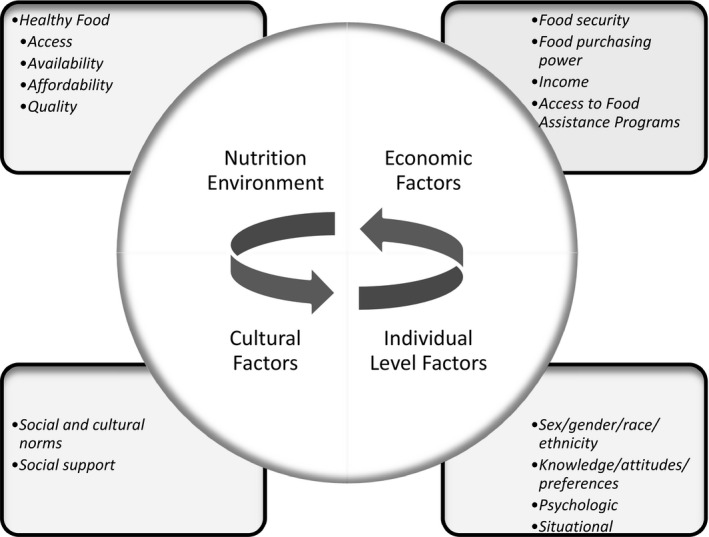
**Determinants of food choice and diet quality.**

The determinants of food choice are complex and have previously been summarized using an adaption of the Social Ecological Model.^35^ As recommended by the 2019 American College of Cardiology/AHA Guideline on the Primary Prevention of CVD, the social determinants of health should inform implementation of treatment recommendations.^4^ The nutrition environment, economic considerations, cultural preferences, and individual‐level factors all contribute to food choice and diet quality, and these factors are likely to interrelate (Figure 2). Some racial/ethnic groups and those of low SES are more likely to experience an unfavorable nutrition environment with suboptimal access to healthy foods. Economic barriers and food insecurity are strong determinates of poor diet quality.^36^, ^37^, ^38^

### Nutrition Environment

Evidence shows neighborhood racial segregation, as a result of historical discriminatory housing policies, is a contributing factor to disparities in CVD risk because fewer disparities exist in more integrated neighborhoods.^39^ Racial segregation is also a major cause of racial disparities in SES, which contributes to the observed disparities in CVD.^40^ In addition, the neighborhood built environment, including the nutrition environment, also influences CVD risk. Historically, racial segregation was accompanied by community disinvestment, which adversely affected the built environment.^40^ As such, unfavorable nutrition environments disproportionately affect some racial/ethnic groups and those of low SES.^41^, ^42^, ^43^, ^44^, ^45^, ^46^ The availability, price, and quality of healthy foods available in a neighborhood relative to less healthy or unhealthy foods directly impact diet quality.

### Food Deserts

Food deserts are defined by the Centers for Disease Control and Prevention as areas that lack access to affordable fruits, vegetables, whole grains, low‐fat milk, and other foods that make up a healthy diet.^47^ The USDA includes low income as part of the food access definition.^48^ Between 2010 and 2015, the number of US census tracts classified as low income increased by 5.4% (29 285 to 30 870).^49^ Food access, defined as proximity to supermarkets, super centers, or grocery stores, improved during the same time frame. Despite this, in 2015, 5.6% to 17.7% of the US population had limited access to a supermarket or grocery store on the basis of proximity measures ranging from 0.5 to 1 mile in urban areas and from 10 to 20 miles in rural areas. However, in low‐income, low‐access census tracts, ≈50% of the population had limited access to supermarkets or grocery stores.

Food deserts disproportionately affect individuals of low‐income, low‐educational attainment and racial/ethnic minorities. In Baltimore, MD, high availability of healthy foods was only present in 19% of predominately black neighborhoods compared with 68% of white neighborhoods.^41^ Furthermore, 46% of low‐income neighborhoods had low availability of healthy foods compared with 18% of high‐income areas. This culminated in white neighborhoods (7.6 points) and high‐income (8.1 points) neighborhoods receiving higher scores on the healthy food availability index compared with predominately black and low‐income areas. Likewise, a study conducted in the Atlanta, GA, metropolitan area found that individuals in food deserts were more likely to be black, were less likely to be college graduates, and had lower income compared with individuals in nonfood deserts.^50^ In addition, living in a food desert was associated with a 14–percentage point higher 10‐year atherosclerotic CVD risk compared with nonfood deserts. Similar disparities in atherosclerotic CVD risk were observed in low‐income areas versus high‐income areas (10–percentage point increase), and for low‐income individuals versus high‐income individuals (15–percentage point increase).^50^

Access to supermarkets and grocery stores stocking affordable healthy foods is associated with greater likelihood of meeting dietary recommendations.^42^ Data from the ARIC (Atherosclerosis Risk in Communities) study cohort, comprising 208 census tracts, demonstrated that NH blacks were more likely to meet recommendations for fruits and vegetables (54% increase in relative risk), total fat (22% increase), and saturated fat (30% increase) if a supermarket was present in their census tract, after adjustment for presence of other types of food stores, income, and education.^42^ A dose‐response relationship existed between supermarket number and intake of fruits and vegetables such that for each supermarket present in a census tract intake increased by 32%. These relationships were less pronounced or nonexistent for NH whites included in the ARIC study sample; this is likely because of the large disparities in supermarket access in predominately black communities. Morland et al^42^ found 5 times more supermarkets were located in census tracts where NH whites lived versus NH blacks.

### Food Swamps

Low‐income areas and communities with a high proportion of racial/ethnic minorities also have a greater density of fast food outlets and convenience stores with meager healthy food offerings.^43^, ^44^, ^45^ The concept of a “food swamp” was coined to describe areas where nutrient‐poor, energy‐dense food availability swamps healthy food options.^51^ Approximately 3.4% to 3.6% of the US population live in a food swamp, defined by the ratio of fast food outlets and convenience stores to supermarkets and grocery stores in a given area.^52^

Typically, the availability, affordability, and quality of healthy foods are greater in supermarkets and grocery stores compared with fast food and convenience stores.^53^ Data from 9 rural counties in Alabama showed healthy foods were available in a greater proportion of grocery stores compared with convenience stores.^53^ For example, 100% of grocery stores stocked some fruit and some vegetables compared with 58% and 78% of convenience stores, respectively. In addition, the vast majority of convenience stores stocked 0 to 4 different fruits and 0 to 9 difference vegetables, whereas all of the grocery stores stocked 5 to 11 different fruits and 10 to 13 different vegetables. Furthermore, the mean price score (0.7 versus 2.4; possible range, −9 to 18; a higher score indicates greater affordability of healthy versus unhealthy foods) and the mean food quality score (0.03 versus 4.2 of 6) were significantly lower for convenience stores than grocery stores.

Living in an area with a high prevalence of fast food outlets and/or a high ratio of fast food outlets and convenience stores to supermarkets and grocery stores alters food choices. In adolescent girls living in Baltimore, MD, from a large urban school district serving predominately low‐income NH black communities, 27% of girls were living in a food desert, and 35% lived in a food swamp, defined as having ≥4 corner/convenience stores within 0.25 miles of their home.^54^ Of the sample, 16% lived in an area classified as both a food desert and a food swamp; ≈54% of the sample lived in neither a food swamp nor a food desert. Living in a food swamp or desert was not associated with consumption of fruits and vegetables, but intake of snacks/desserts was greater in food swamps (3.71 servings/day) compared with nonfood swamps (3.07 servings/day). In addition, living in both a food swamp and a food desert was related to greater consumption of snacks/desserts compared with living in an area that was neither a food swamp nor a food dessert (3.81 versus 3.22 servings/day).

Neighborhood data from the CARDIA (Coronary Artery Risk Development in Young Adults) study, including Birmingham, AL, Chicago, IL, Minneapolis, MN, and Oakland, CA, showed a relationship between the racial and income status of a neighborhood and the density of fast food offerings.^55^ Rummo et al^55^ found that for every 1% increase in the percentage of the white population, there was a 17% decrease in the density of fast food outlets in low‐income neighborhoods; however, in high‐income neighborhoods, a 22% increase in fast food outlets was observed. A subsequent analysis of this cohort showed that a 1% increase in fast food availability within <1 km or 1 to 2.9 km of respondents’ homes was associated with a 0.13% and 0.34% increase in fast food consumption frequency in low‐income men, respectively.^57^

### Economic Barriers: Food Insecurity

Food insecurity is defined by the USDA as reduced quality, variety, or desirability of the foods and diets consumed with/without reduced food intake.^58^ Low food security is defined as multiple indications of disrupted eating patterns and reduced food intake. Vercammen et al^59^ reported that those with low food security had more than twice the odds of presenting with a 10‐year CVD risk of ≥20% (odds ratio, 2.36; 95% CI, 1.25–4.46) compared with food secure individuals. In 2017, ≈12% of US households were food insecure at some point during the year, and 4.5% of these households had low food security.^60^ Households with any children (15.7%), children aged <6 years (16.4%), headed by single women (30.3%) or men (19.7%), headed by NH blacks (21.8%) or Hispanics (18%), or income <185% of the poverty threshold (30.8%) were disproportionately affected by food insecurity. An analysis of the 2016 Medicare Current Beneficiary Survey found that 38.3% of enrollees aged <65 years and 9.1% of enrollees aged >65 years were food insecure, and a greater burden of food insecurity was observed in those of low income in both age ranges.^56^ On average, food insecure households spend 23% less on food than a comparable food secure household of the same size and composition.^60^

Food insecurity or limited access to adequate food is attributable to economic factors, including food pricing and income level, and food availability and access. The Thrifty Food Plan (TFP), last revised in 2006, is a low‐cost meal plan that aligns with the 2005 Dietary Guidelines for Americans and the 2005 MyPyramid Food Guidance System.^61^ Although TFP is the basis for the maximum SNAP allocation and is the national standard for a nutritious diet at minimal financial cost, data show it is not a realistic estimate of the cost of a diet meeting minimum daily dietary requirements. Age‐sex stratified TFP market baskets are developed using a complex algorithm that includes average consumption patterns of low‐income individuals (by food category), cost (per 100 g of each food category), nutrient profile (per 100 g of each food category), and the MyPyramid profile (per 100 g of each food category).^61^ The algorithm is constrained by dietary standards (2005 MyPyramid food intake recommendations, the 2005 Dietary Guidelines for Americans, and the 1997–2005 Recommended Dietary Allowances) and TFP maximum cost allotment (inflation‐adjusted average cost of the previous iteration). A systematic review of 16 market basket surveys conducted in the United States between 1985 and 2012 showed that, in nearly all of the studies (10/11), the cost of a market basket meeting minimum daily dietary requirements at a small/medium store was greater than the TFP.^62^ Furthermore, 60% of studies (9/15) found that a market basket was above the TFP at supermarkets, and 3 studies (of 7) reported costs above the TFP in low‐income neighborhoods.

The TFP is based on the assumption that all foods are purchased, prepared from raw ingredients, and consumed at home. Estimates suggest weekly meal plans, according to the recipes in the TFP, require 2.3 h/d (~16 h/wk) of cooking and preparation time, which is counter to contemporary trends in time spent preparing food.^63^ In 2018, on average, Americans spent 0.6 h/d on food preparation and cleanup.^64^ Two economic analyses report that the average single‐headed household spends 34% to 35% more on food than allocated by the TFP, and 62% of households spend enough to meet the TFP.^65^, ^66^ However, when the time required for food preparation is factored into the estimates, single‐headed households spend 40% less than needed to meet the TFP, and only 13% of households spend enough to meet the TFP.^65^ These analyses suggest that the labor burden associated with meeting the TFP needs to be revised to align with current estimates of the time spent preparing food and potentially a greater financial supplement is required to enable purchasing of ingredients and foods that require less food preparation time. Future iterations of the TFP should consider total resources required to meet the weekly meal plans.^67^

Although SNAP increases food purchasing power, allocations may not reach a level that ensures food security.^68^, ^69^, ^70^ Estimates from 2011 to 2012 suggest SNAP receipt reduces food insecurity by 6% to 17% and low food security by 12% to 19%.^70^ However, not all SNAP eligible households choose to participate in SNAP,^71^ and the rate of nonparticipation is difficult to accurately ascertain because of misreporting, but the USDA reported that 85% of eligible individuals were served by SNAP in 2016, although participation was as low as 30% for those eligible for minimum benefit and 42% for elderly individuals.^72^ The most common reasons for nonparticipation are stigma surrounding the receipt of benefits, transaction costs for SNAP recertification, and eligibility for minimum or low‐benefit only.^71^

Food insecurity is associated with worse diet quality^36^, ^37^, ^38^; a National Health and Nutrition Examination Survey analysis (2011–2014) showed that, compared with food secure adults, food insecure adults had a 2.22‐point lower HEI‐2015 score.^36^ Some evidence suggests that food insecurity may result in worse diet quality in white individuals compared with black individuals for reasons that are not clear.^36^, ^73^ However, in both the National Health and Nutrition Examination Survey analysis by Leung et al^36^ and the Healthy Aging in Neighborhoods of Diversity Across the Life Span (Baltimore, MD)^73^ analysis, it was found that NH blacks were more likely to be food insecure compared with NH whites. Furthermore, Allen et al^73^ reported that NH blacks were more likely to participate in SNAP, compared with food insecure NH whites, and SNAP participation diminished the effect of food insecurity on diet quality in NH blacks only. Therefore, complex interrelations exist between race/ethnicity, food insecurity, food assistance participation, and diet quality, and further research is required to understand the multidimensional determinants of food insecurity and diet quality by race/ethnicity.

Disparities in diet quality by food security status are likely explained by the positive linear association observed between diet cost and diet quality in analyses of population consumption patterns.^74^ A meta‐analysis of 27 studies, representing 10 countries, found a healthy dietary pattern costs more, on average, by a magnitude of $1.48/day per person or $1.54/2000 kcal.^75^ Recent estimates suggest a diet consistent with the 2015 to 2020 Dietary Guidelines for Americans recommended healthy eating patterns would cost $8.27/day per person for the Healthy US Style Eating Pattern, $8.73/day per person for the Healthy Mediterranean‐Style Eating Pattern, and $5.90/day per person for the Healthy Vegetarian Eating Pattern.^76^ However, actual spending on food per day was lower for all racial/ethnic groups on the basis of reported dietary intake in National Health and Nutrition Examination Survey 2013 to 2014 (Hispanics spent $5.47/day; NH blacks spent $5.45/day; NH whites spent $5.94/day; and NH Asians spent $6.57/day).

## Food Assistance Programs for Underserved Populations

Federal food assistance programs, including SNAP, the Special Supplemental Nutrition Program for Women, Infants, and Children (WIC), and school meal programs exist to augment recipients’ food purchasing power. In 2017, ≈58% of food insecure households participated in ≥1 of these federally funded food assistance programs.^60^ SNAP, American's largest nutrition assistance program, serves ≈1 in 7 Americans; ≈25% of recipients are NH blacks, 36% are NH whites, and 17% are Hispanics.^77^ SNAP benefits apply to any food product, except for alcoholic beverages, food for animals, lunch‐counter items, foods to be eaten in stores, and vitamins. In some jurisdictions, SNAP benefits may be used at restaurants. The major eligibility criterion for SNAP is a gross income of ≤130% of the federal poverty threshold, although several other criteria are assessed in the SNAP eligibility process.^78^

In 2009, WIC was revised to include more fruits, vegetables, whole grains, and lower‐fat milk; this was associated with a significant improvement in HEI‐2010 score (3.7 points; 95% CI, 0.6–6.9).^2^ Unlike the WIC program, SNAP does not regulate the foods that can be purchased with SNAP dollars. Some have suggested that WIC‐style restrictions should be applied to SNAP to improve diet quality and improve health outcomes.^79^ However, this is particularly polarizing and many issues surrounding the feasibility and justification for implementation, likely effectiveness, the definition of “unhealthy” foods, and how the classifications will be consistently applied, and the preservation of the dignity of recipients have been debated.^80^

USDA data suggest that SNAP and non‐SNAP households have relatively similar food purchasing habits,^81^ although an analysis of transactions at a large supermarket chain in the Northeastern United States showed that SNAP transactions included a higher percentage of sales of sugar‐sweetened beverages, red meat, processed meat, pizza, and cold convenience food, and a lower percentage of sales of fruits, vegetables, nuts, and seeds.^82^ Furthermore, a longitudinal analysis of ≈60 000 households’ purchase data found that SNAP participants purchase fewer fruits and less fiber, and more processed meats, sweeteners and toppings, sugar‐sweetened beverages, total energy, sodium, and sugar compared with eligible nonparticipants and ineligible nonparticipants.^83^ However, often low‐income individuals base their purchasing decisions on price per calorie^84^ and typically energy‐dense (often nutrient poor) foods cost less on a price per calorie basis.^85^, ^86^, ^87^, ^88^ Thus, to improve the diets of underserved populations, strategies that increase purchasing power for healthy foods and reduce the affordability of unhealthy foods are needed.

## Public Policy Changes to Improve Diet Quality and Cardiovascular Health in Underserved Populations

As discussed in the previous section, many social and environmental factors influence diet quality and CVD risk; thus, public policies addressing these root causes are critical to reduce the burden of diet‐related CVD. As depicted by Frieden in the Public Health Pyramid, interventions that target socioeconomic factors have the greatest impact on population health and require the least individual effort,^89^, ^90^ therefore increasing the likelihood that the easiest choice is the healthiest choice. Effective policies to improve diet target: (1) the nutrition environment, including the availability of supermarkets and other places where healthy, affordable food can be purchased; (2) affordability of healthy foods, including economic incentives (eg, subsidies for healthy foods and taxes on unhealthy foods); (3) the food environment in public places, such as schools, universities, workplaces, or healthcare settings, including availability and access to healthy foods, education, and incentives for healthy eating; (4) marketing of foods and beverages, including standards for advertising to children and vulnerable populations; and (5) food labeling, including mandating the nutrition facts panel and/or other information that must appear on food packages to inform consumers and to influence industry product formulations.^91^, ^92^, ^93^ Empirical evidence for the effectiveness of these types of policies is often difficult to ascertain^93^ (eg, implementation and evaluation research for policies targeting the nutrition environment is lacking).^94^ However, the likely impact of financial incentives in low‐income groups has been quantified.

Financial incentives are effective modulators of behavior change.^95^, ^96^ Therefore, offering financial incentives to encourage purchasing of healthy foods and/or disincentives or restrictions on purchasing of unhealthy foods may improve diet quality, especially in low‐income groups and SNAP participants. A systematic review and meta‐analysis of prospective studies showed that a 10% reduction (subsidy) in the price of healthy foods increased consumption by 12%; intake of fruits and vegetables increased by 14%.^96^ Moreover, an increase in the cost (tax) of unhealthy foods decreased consumption by 6%. This approach reduced intake of sugar‐sweetened beverages (9%), fast food (3%), and other unhealthy beverages (5%).

On the basis of modeling estimates, public policy to nationally reduce the cost (subsidy) of fruits, vegetables, nuts, and whole grains by 10% is expected to increase consumption of these foods in the whole population (ie, SNAP eligible participants, SNAP eligible nonparticipants, and higher‐income SNAP ineligible individuals). This scenario is projected to reduce deaths from CHD by ≈2.2%.^97^ Furthermore, a 10% increase (tax) in the national cost of sugar‐sweetened beverages and processed meats would be expected to reduce consumption population wide; however, greater reductions would be observed in SNAP participants versus SNAP nonparticipants (eligible and ineligible). This would result in more CHD deaths being averted in SNAP participants versus eligible and ineligible nonparticipants (4.3% versus 2.9%–3.5%). If in addition to the 10% subsidy and 10% tax, SNAP participants received a 30% reduction in the cost of fruits, vegetables, nuts, and whole grains, greater reductions in CHD death would be realized in SNAP participants (8%) versus SNAP eligible nonparticipants (3.5%) and SNAP ineligible nonparticipants (2.9%). These analyses are based on theoretical estimates of the likely impact of subsidies/taxes on categories of food for SNAP participants and nonparticipants; however, in reality, this is likely more nuanced and implementation will likely be challenging because of issues such as the definition of foods eligible for subsidies/tax and the potential for unintended consequences (eg, undesirable shifts in diet).

Financial incentives and disincentives have been evaluated in 3 intervention studies.^98^, ^99^, ^100^ The USDA Healthy Incentives Pilot program, conducted in a nonrepresentative sample of SNAP participants in Hampden Country, MA, demonstrated that a 30% cash incentive for targeted fruit and vegetable purchases increased fruit and vegetable consumption by about one‐quarter cup‐equivalents per day (or 26%) over a 12‐month period.^98^ The incentive also resulted in a 4.7‐point increase (or 8%) in the HEI‐2010 score compared with the control group (*P*<0.001). In a similar randomized, controlled, pilot study, the HDS (Healthy Double Study), rural SNAP and non‐SNAP participants shopping at a supermarket chain in Portland, ME, received 2 for 1 discounts on targeted fruits and vegetables in the form of a coupon provided at the checkout.^99^ After 3 months, a greater amount of weekly spending was attributed to fruits and vegetables in the intervention group compared with the controls not receiving discounts. Furthermore, greater increases in fruit and vegetable spending were observed in SNAP participants versus nonparticipants. In another study, low‐income adults not enrolled in SNAP from Minneapolis–St Paul, MN, were randomized to receive: (1) a 30% financial incentive for purchasing fruits and vegetables; (2) restriction placed on food benefits so no sugar sweetened beverages, sweet baked goods, or candies could be purchased with food benefits; (3) condition 1 plus 2 (incentive+restriction); or (4) control (no restriction or incentive).^100^ After 12 weeks, in comparison to the control condition, increases in the HEI‐2010 score were observed in both the incentive plus restriction condition (4.1 points) and the incentive only condition (1.6 points), *P*<0.05, which was mostly attributed to reductions in sugar‐sweetened beverages and intake of all the restricted foods. These pilot studies suggest that financial incentives may assist in achieving dietary improvements, particularly increasing fruit and vegetable consumption; however, the findings need to be confirmed in larger trials conducted in cohorts representative of low‐income populations who would be targeted by financial incentive/disincentive policies.

Using a nationally representative data set and based on data from the Healthy Incentives Pilot, it was estimated that a 30% incentive for fruit and vegetable purchases would modestly increase fruit (~19 g/d; ~23%) and vegetable (~26 g/d; 19%) consumption in SNAP participants, which would result in 303 911 CVD deaths being averted and 649 376 quality‐adjusted life years would be gained over a lifetime; the projected societal cost saving is $6.69 billion.^101^ Coupling the 30% incentive for fruit and vegetable purchases with a restriction on purchase of sugar‐sweetened beverages with SNAP dollars would be expected to reduce consumption of sugar‐sweetened beverages by 139 g/d (33%), and approximately double to quadruple the health gains (797 888 CVD deaths would be averted and 2 106 832 quality‐adjusted life years would be gained with a projected societal cost saving of $39.05 billion). Finally, 30% incentives for fruits and vegetables, nuts, whole grains, fish, and liquid oils, and 30% disincentives for sugar‐sweetened beverages, junk food, and processed meats were modeled. This scenario was estimated to increase consumption of healthy foods by 19% to 24% and reduce intake of unhealthy foods by 13% to 17%, resulting in 939 965 CVD cases being avoided, 2 465 008 quality‐adjusted life years gained, and societal cost savings of $41.82 billion. In terms of cost‐effectiveness, all 3 of the proposed changes to the SNAP system met traditional cost‐effectiveness thresholds over a lifetime.

These findings from modeling analyses and pilot research suggest that financial incentives for use of SNAP dollars for healthful foods and disincentives or restriction on use for nutrient‐poor, energy‐dense foods may benefit diet quality, as well as cardiovascular health, although further implementation research in populations representative of those who would be targeted by changes to the SNAP system is required to completely understand the impact. On the basis of the available evidence, it is expected that these modifications would alleviate economic barriers that reduce the capacity for purchasing and preparation of healthy meals. In addition, these changes would likely improve adherence to the TFP by reducing the financial cost of foods and encourage more healthful purchasing patterns that may assist with reducing diet quality disparities and achievement of health gains, especially for CVD in low‐income SNAP participants. There is also some support for changes to SNAP from stakeholders and program participants.^102^, ^103^ A 2013 survey of SNAP participants in California showed overwhelming support for program changes to improve nutritional quality, including restrictions on sugar‐sweetened beverages and monetary incentives for fruits and vegetable purchases.^103^ However, from a policy perspective, significant innovations to the SNAP program to encourage healthier diets are needed now, but modifications typically are incremental or modest and, consequently, limit the public health gains that could be realized.^104^

## Community and Organizational Programs to Improve Diet Quality and Cardiovascular Health in Underserved Populations

Several community and organizational programs exist to improve diet and CVD health in underserved populations. These programs are typically focused on improving healthy food availability and access, food literacy, and nutrition knowledge. The most established are the federally funded nutrition education programs that are administered at a state agency level. The Expanded Food and Nutrition Education Program (EFNEP) is the first US education program targeted at low‐income populations.^105^ This program operates in all US states, the District of Columbia, and the 6 US territories (American Samoa, Guam, Micronesia, Northern Marianas, Puerto Rico, and the Virgin Islands) with the goal of using education to support self‐sufficiency, nutritional health, and well‐being. A preevaluation/postevaluation of Maine EFNEP participants from 2013 to 2016 (n=507) showed diet quality, measured by HEI‐2005, increased from 52.6 points (of 100) to 59 points after the program, and participants who spent <7 hours in the program had a smaller improvement in diet quality versus those who spent 7 to 16 hours (4.65 versus 8.44 points; *P*=0.05).^106^ In this sample, ~70% received SNAP, 51% were enrolled in WIC, and 37% were using both, and the majority (>85%) of the sample were NH white and women. In a similar analysis of predominantly Hispanic (61%) or NH white (28%) women participating in EFNEP from 8 states of the US mountains region from 2010 to 2011 (n=3338), an increase in diet quality, measured by HEI‐2005, from 49.4 (interquartile range, 39.9–58.7) to 55.7 (interquartile range, 45.8–65.0) was observed after median participation in 6 EFNEP lessons.^107^ In this analysis, the daily cost of diet increased from $3.94 (interquartile range, $2.75–$3.27) at program entry to $4.44 (interquartile range, $5.44–$5.87) at program exit, a 13% increase. Participation in other food assistance programs was not reported in this study. These data suggest EFNEP increases diet quality in program participants, although relatively few analyses have been conducted and the cohorts studied may not be representative of the broader EFNEP target population, indicating the need for further research and evaluation.

SNAP‐Education (SNAP‐Ed) is another federally funded nutrition education program, complementary to SNAP, administered by agencies at a state level.^108^ The goal of SNAP‐Ed is to use community/public health approaches to provide educational programs, messaging, policies, and systems to increase food security and adherence to the Dietary Guidelines. To be eligible for SNAP‐Ed, individuals do not have to participate in SNAP, but this is the target population. Relatively few quantitative analyses have assessed the impact of SNAP‐Ed on diet quality^109^; however, some evidence from relatively small trials shows SNAP‐Ed improves food security during the education period (4–7 weeks),^110^, ^111^, ^112^ although the longer‐term impacts are less clear.^109^ In the only randomized controlled trial to collect longer‐term outcome data, household food security improved by 25% (or 1.2±0.4 units on the US Household Food Security Scale) after 1 year in low‐income households in Indiana with at least 1 child, after participation in 4 to 10 weeks of SNAP‐Ed, compared with a control group.^113^ There is currently inconsistent and limited evidence to suggest SNAP‐Ed improves dietary intake,^109^ although this is primarily because of the lack of rigorous evaluation of the program, which underscores the importance of research in this area to quantify the impact and potential for changes to promote effectiveness.

Several other community or organizational level programs aimed at increasing food literacy and improving diet quality have been implemented and in some cases evaluated. For example, the Healthier Options for Public School program, in Florida, implemented a nutrition curriculum and modified school meals, and observed significant reductions in overall weight among girls with a trend toward reduced blood pressure over a 2‐year follow‐up period.^114^ In Los Angeles, CA, farm‐to‐school strategies are used for nutrition education and to facilitate delivery of farm fresh fruits and vegetables to school children.^115^ Similarly, in Minnesota, the Youth Farm and Market Project was designed to teach inner‐city youth hands‐on gardening and cooking as part of nutrition education.^116^ The AHA Simple Cooking With Heart Kitchen initiative provides hands‐on instruction on preparing affordable, healthy foods using standard cooking equipment and easily accessible and inexpensive ingredients.^117^ Other innovative approaches to improve nutrition for low‐income groups include the addition of neighborhood farm stands and farmers’ markets.^118^, ^119^ Recent data indicate that supporting farmers’ market incentive programs translates not only into better food security for underserved, low‐income households but also into greater consumption of fresh vegetables.^120^

## Multilevel Interventions With a Dietary Component to Address Disparities Associated With CVD

With recognition of the complex, multilevel factors that influence the presence of disparities in CVD, since 2003, the National Institutes of Health has funded 10 Centers for Population Health and Health Disparities across the country to develop and implement interventions and programs to address disparities at individual, household, and organization levels (Table). Although most of these interventions are multifaceted in nature, all included at least one critical dietary component, such as individual‐level nutritional education,^121^, ^122^, ^123^ dietary behavior–change training,^122^, ^124^, ^125^, ^126^ weight loss interventions,^125^ or corner store interventions to improve access to healthy foods.^127^ In general, positive impacts on healthy eating were observed^123^, ^124^, ^125^, ^126^ and a few studies suggested potential beneficial effects of the interventions on blood pressure control.^121^, ^125^ However, this evidence base is limited by the small sample sizes of the studies and the lack of detail on the sustainability of the interventions in terms of long‐term behavior modification and CVD outcomes.

**Table 1:** Multilevel Interventions Including a Dietary Component to Address Disparities Associated With CVD

Project	Population (Location)	No. of Participants	Intervention Duration	Multilevel Intervention	Dietary Component of the Intervention	Results
CHART (Congestive Heart Failure Redesign Trial: A Pilot Study)^124^	Low‐income patients hospitalized with heart failure (Chicago, IL)	n=20 Physicians; n=33 patients (23 completed the study)	4 mo	Culturally sensitive, multilevel long‐term care intervention to improve self‐management behaviors by providing patient and provider education; community healthcare worker intervention	Patients received self‐management training for diet by a trained nurse through 11 interactive sessions over a 4‐mo period	Median sodium intake declined (3.5 vs 2.0 g/d; *P*<0.01)
Heart Healthy Lenoir Project—Lifestyle study^125^	Patients receiving hypertension care in rural primary care practices (Lenoir County, North Carolina)	339	2 y	Phase 1: improving diet quality and increasing physical activity (6 mo); Phase 2: weight loss intervention for patients with a BMI ≥25 kg/m^2^ or a lifestyle maintenance intervention for patients with a BMI <25 kg/m^2^ (6 mo); Phase 3: RCT comparing intensive with less intensive weight loss maintenance intervention for subjects who lost ≥8 lb in phase 2 or lifestyle maintenance intervention for all other subjects (1 y)	See left	Phase 1 (n=251 completed). Substantial improvement in diet score (4.3 units), systolic BP (−6.4 mm Hg; −8.7 to −4.1 mm Hg) that was maintained for 2‐y follow‐up; Phase 2 (n=138): weight change was −3.1 kg (−4.9 to −1.3) for group sessions and −2.1 kg (−3.2 to −1.0) for group/telephone session; Phase 3 (n=24): weight loss was −2.1 (−4.3 to 0.0) and −1.1 kg (−2.7 to 0.4) for the 2 groups, respectively. Outcomes for blacks and whites were similar: there was a substantial improvement in diet and BP, but weight loss was modest
Project Red Chip (Reducing Disparities and Controlling Hypertension in Primary Care)^121^	Six primary care clinics in predominantly black urban communities (4 were located in medically underserved areas in Baltimore, MD)	3964 Uncontrolled hypertensive patients	3 mo	Multimethod, staged quality improvement intervention (targeting better BP measurement; patient case management; provider education, including audit and feedback and communication skills training)	Patients received 3 care management educational sessions (totaling 120 min), including lifestyle behaviors covered by RDs related to DASH diet, weight loss, and exercise	Those completing all sessions (629 participated in education; 245 completed all 3 sessions) on average reached BP control (mean systolic/diastolic BP, 137/78 mm Hg) and systolic BP was reduced by 9 mm Hg (*P*<0.001) and 4 mm Hg diastolic BP (*P*=0.004) greater improvement than nonparticipants; Disparities in systolic and diastolic BP between blacks and whites were no longer present at the end of the study. However, the limited reach indicated that care management alone may not improve BP control and eliminate disparities
ACT (Achieving Blood Pressure Control Together) Study^122^	Black adults with uncontrolled hypertension in a primary care clinic in a low‐income area (Baltimore, MD)	159	12 mo	1) An educational intervention led by a community health worker alone, 2) the community health worker intervention plus a patient and family communication activation intervention, or 3) the community health worker intervention plus a problem‐solving intervention	The community health worker intervention reviewed and reinforced education on high BP, nutrition, and exercise. In the problem‐solving intervention, participants learned about behavioral goals for monitoring their BP and making effective diet and lifestyle modifications for BP control	No results published yet
Proyecto Mercado Fresco (fresh market project)^127^	Predominantly Latino communities (East Los Angeles, CA, and Boyle Heights, CA)	n=4 Corner stores	2 y	The project used a community‐engaged approach to select, recruit, and convert 4 corner stores to improve access to and awareness of fresh affordable fruits and vegetables. This is a multilevel corner store conversion intervention, which includes having multiple stakeholders, expertise in corner store operations, community and youth engagement strategies, and social marketing campaigns	See left	Improvements were found in perceived healthy food accessibility and perceptions of corner stores. No changes were found, however, in store patronage, purchasing, or consumption of fruits and vegetables
North Carolina Wisewomen Project,^123^	Low‐income women (31 counties in North Carolina)	n=17 Counties’ health department in the MI group; n=14 in the EI group; n=2148 women screened	6 mo	Expanding an existing cancer screening program to include a CVD screening and intervention program. The enhanced intervention included 3 specially constructed counseling sessions spanning 6 mo using a structured assessment and intervention program tailored to lower‐income women	The counseling sessions included diet and physical activity using a tailored, culturally appropriate, structured assessment and intervention program for patients with lower literacy and low income. The program is known as “A New Leaf”	After 6 mo of follow‐up in the EI health departments, changes in total cholesterol levels, HDL‐C levels, diastolic BP, and BMI were observed (−5.8 mg/dL, −0.9 mg/dL, −1.7 mm Hg, and −0.3 kg/m^2^, respectively), but were not significantly different from MI health departments. A dietary score that summarized fat and cholesterol intake improved by 2.1 units in the EI group, compared with essentially no change in the MI group
Five Plus Nuts and Beans trial^126^	Blacks with controlled hypertension from an urban primary care clinic (Baltimore, MD)	123	8 wk	A program providing dietary advice and assistance with grocery ordering, and $30 per week of high‐potassium foods	In the intervention group, known as the DASH‐Plus group, subjects received coach‐directed dietary advice and assistance with weekly online ordering and purchasing of high‐potassium foods delivered by community supermarkets to a neighborhood library	Compared with the control group, the DASH‐Plus group increased self‐reported consumption of fruits and vegetables (mean, 1.4; 95% CI, 0.7–2.1 servings/d); estimated intake of potassium (mean, 0.4; 95% CI, 0.1–0.7 g/d); and urine potassium excretion (mean, 19%; 95% CI, 1%–38%). There was no significant effect on BP

BMI indicates body mass index; BP, blood pressure; CVD, cardiovascular disease; DASH, Dietary Approaches to Stop Hypertension; EI, enhanced intervention; HDL‐C, high‐density lipoprotein cholesterol; MI, minimum intervention; RCT, randomized controlled trial; and RD, registered dietitian.

## A Call to Action

The challenge to reduce and ideally eliminate health inequities is complex and was extensively analyzed after the Institute of Medicine report, Unequal Treatment: Confronting Racial and Ethnic Disparities in Health Care in 2002.^128^ Underserved populations have poorer health outcomes, and despite robust research evaluating various interventions, these disparities persist. Multifaceted approaches that strategically address the social determinants of diet quality and CVD, including confronting barriers to a healthy diet in underserved populations, are essential to support public health policies and foster effective community‐level intervention^47^.

## Conclusions

In the United States, disparities in diet quality exist by race/ethnicity and SES, which mirrors disparities in CVD burden. There is also a conjoined need to address the prevailing suboptimal quality of the US diet and its implications for cardiovascular health population wide. Barriers to a healthy diet at the government (policy), community environment, sociocultural, and individual levels have been summarized, and areas requiring action have been highlighted. Evidence suggests that revisions to public policy to increase the affordability of healthy foods may benefit low‐income groups and those using food assistance programs most significantly. Modifications to SNAP to incentivize purchasing of healthy foods could increase SNAP recipient's healthy food purchasing power, and disincentivize or restrict the use of SNAP dollars for unhealthy food purchases with a resultant benefit on diet quality, as well as CVD health. However, much remains to be done to improve population diet quality, especially in populations who are disproportionally affected by an unfavorable nutrition environment and economic barriers.

## Disclosures

Dr Miller serves as a Trustee for the AstraZeneca HealthCare Foundation, Connections for Cardiovascular Health (CHC). He receives no compensation, has not applied for funding through CHC, and is recused from participating in University of Maryland proposals. Dr Freeman does nonpromotional speaking for Boehringer‐Ingleheim. The remaining authors have no disclosures to report.

## Supporting information


**Table S1**
**Figures S1–S4Reference 11**
