# Supplementary material for: Barriers, Opportunities, and Challenges in Addressing Disparities in Diet‐Related Cardiovascular Disease in the United States
Source: J Am Heart Assoc. 2020 Mar 23;9(7):e014433. doi: 10.1161/JAHA.119.014433 (PMC7428614; doi:10.1161/JAHA.119.014433)
Supplement: Supplementary file 1 — Table S1 Figures S1–S4Reference 11 [file JAH3-9-e014433-s001.pdf]

# Supplemental Material

**Table S1. Ideal, Intermediate and Poor Levels of AHA's Life's Simple 7 Components.**

| Component | Ideal | Intermediate | Poor |
|---|---|---|---|
| Smoking | Never or former > 1 year | Former ≤1 year | Current |
| Healthy Diet Score<br>*Primary Components**<br>*Fruits and vegetables*<br>*Fish 3.5-oz servings (preferably oily fish)*<br>*Sodium*<br>*Sweets/sugar-sweetened Beverages*<br>*Whole grains, 1-oz-equivalent servings* | 4-5 components<br><br>*≥ 4.5 cups/day*<br>*≥2 servings/week*<br><br>*1500 mg/day*<br>*≤450 kcal (36 oz.)/week*<br><br>*≥3 servings/day* | 2-3 components | 0-1 components |
| Physical Activity | ≥150 min/week moderate intensity or ≥75 min/week vigorous intensity or ≥150 min/week moderate + vigorous | 1–149 min/week moderate intensity or 1–74 min/week vigorous intensity or 1–149 min/week moderate + vigorous | None |
| Body Mass Index | < 25 $kg/m^2$ | 25-29.9 $kg/m^2$ | ≥30 $kg/m^2$ |
| Blood Pressure | <120/<80 mm Hg untreated | SBP 120-139 or DBP 80-89 mmHg or treated to ideal level | SBP ≥140 or DBP ≥90 mmHg |
| Total Cholesterol | <5.18 mmol/L (<200 mg/dL) untreated | 5.18-6.19 mmol/L (200-239 mg/dL) or treated to ideal level | ≥6.22 mmol/L (≥240 mg/dL) |
| Fasting Glucose | <5.55 mmol/L (<100 mg/dL) untreated | 5.55-6.94 mmol/L (100-125 mg/dL) or treated to ideal level | ≥6.99 mmol/L (≥126 mg/dL) |

*Scaled for 2000 kcal/day; DBP, diastolic blood pressure; SBP, systolic blood pressure.

Adapted from[11]

**Figure S1. Mean Dietary Intakes for Primary Components of the American Heart Association's Healthy Diet Score by Race/Ethnicity and Age Group: NHANES 2015-2016.**

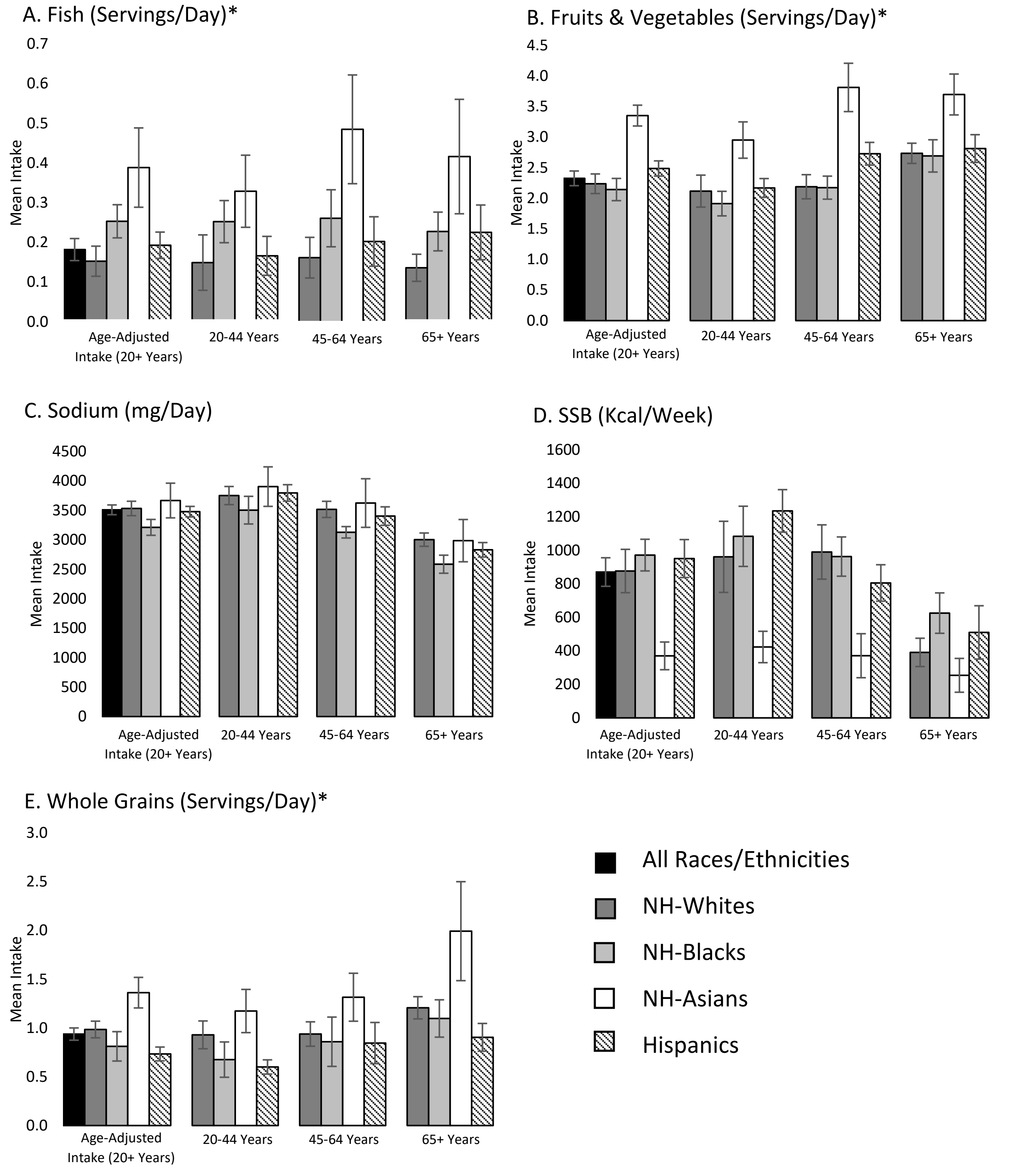


*Intakes standardized to a 2,000 kcal/day diet; Error bars represent 95% Confidence Intervals

**Figure S2. Mean Dietary Intakes for Primary Components of the American Heart Association's Healthy Diet Score by Income to Poverty Ratio (IPR) and Age Group: NHANES 2015-2016.**

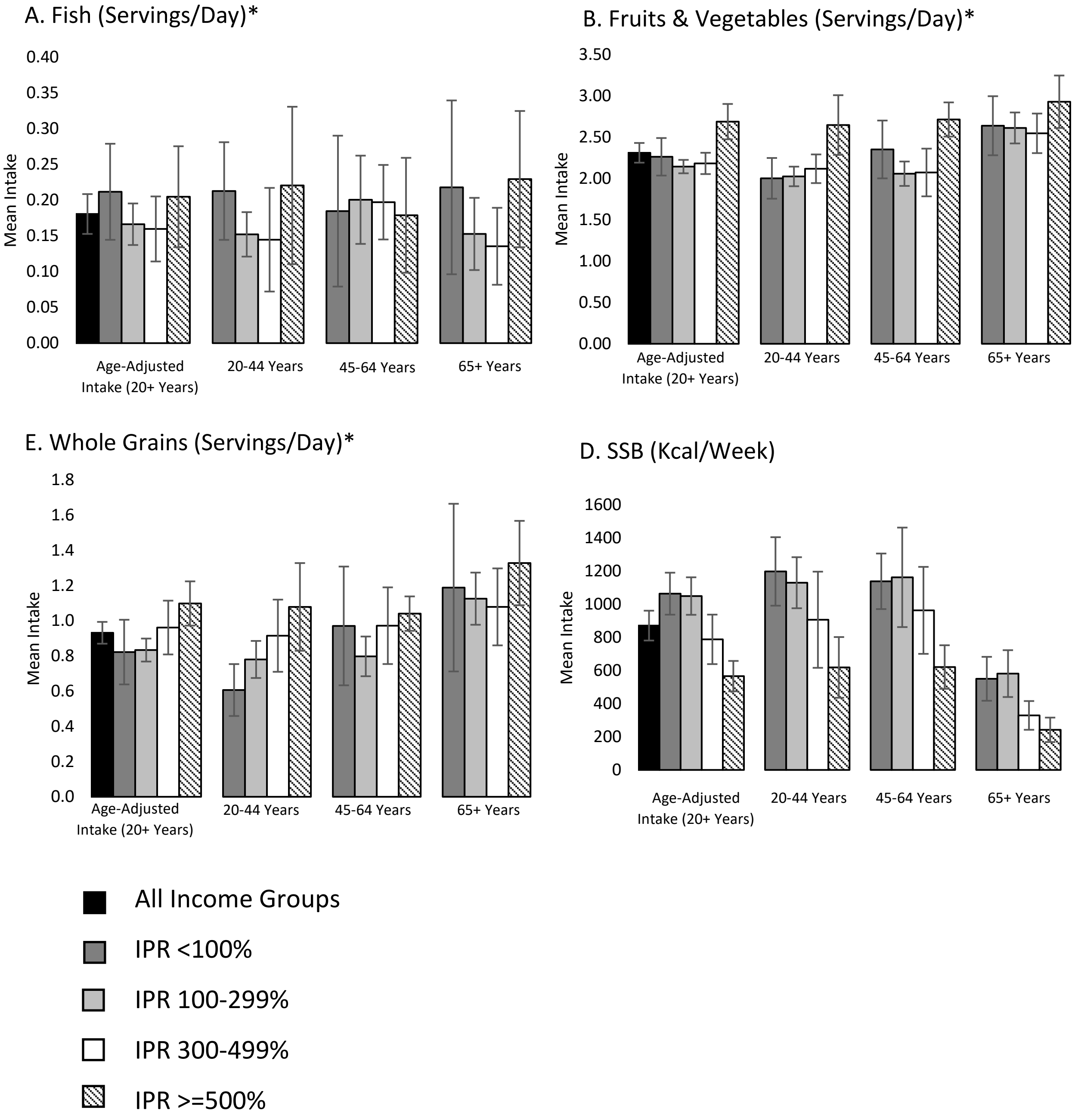


*Intakes standardized to a 2,000 kcal/day diet; Error bars represent 95% Confidence Intervals

**Figure S3. Mean Dietary Intakes for Primary Components of the American Heart Association's Healthy Diet Score by Educational Attainment and Age Group: NHANES 2015-2016.**

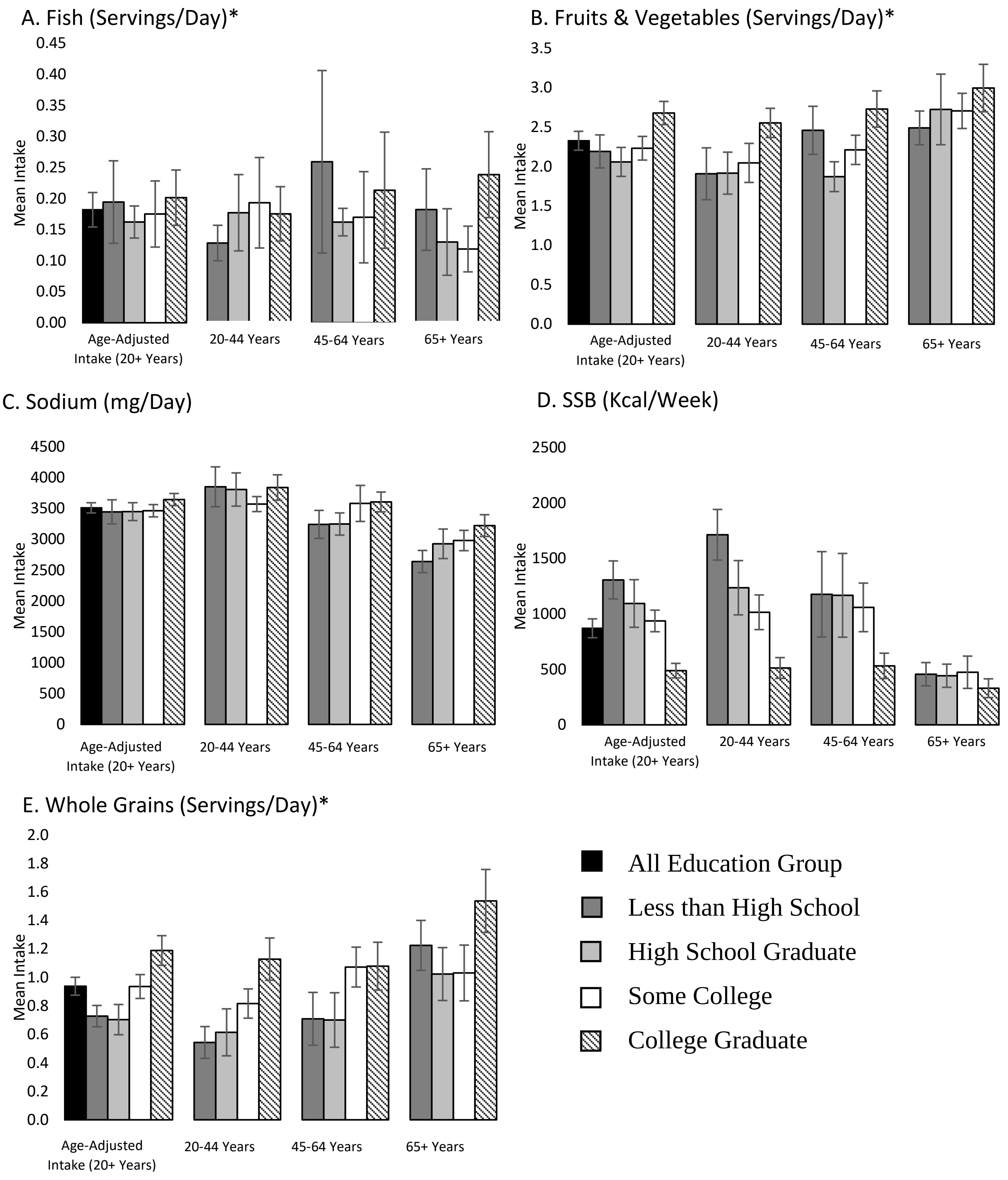


*Intakes standardized to a 2,000 kcal/day diet; Error bars represent 95% Confidence Intervals

**Figure S4: Mean Dietary Intakes for Primary Components of the American Heart Association's Healthy Diet Score by Health Insurance Type and Age Group: NHANES 2015-2016.**

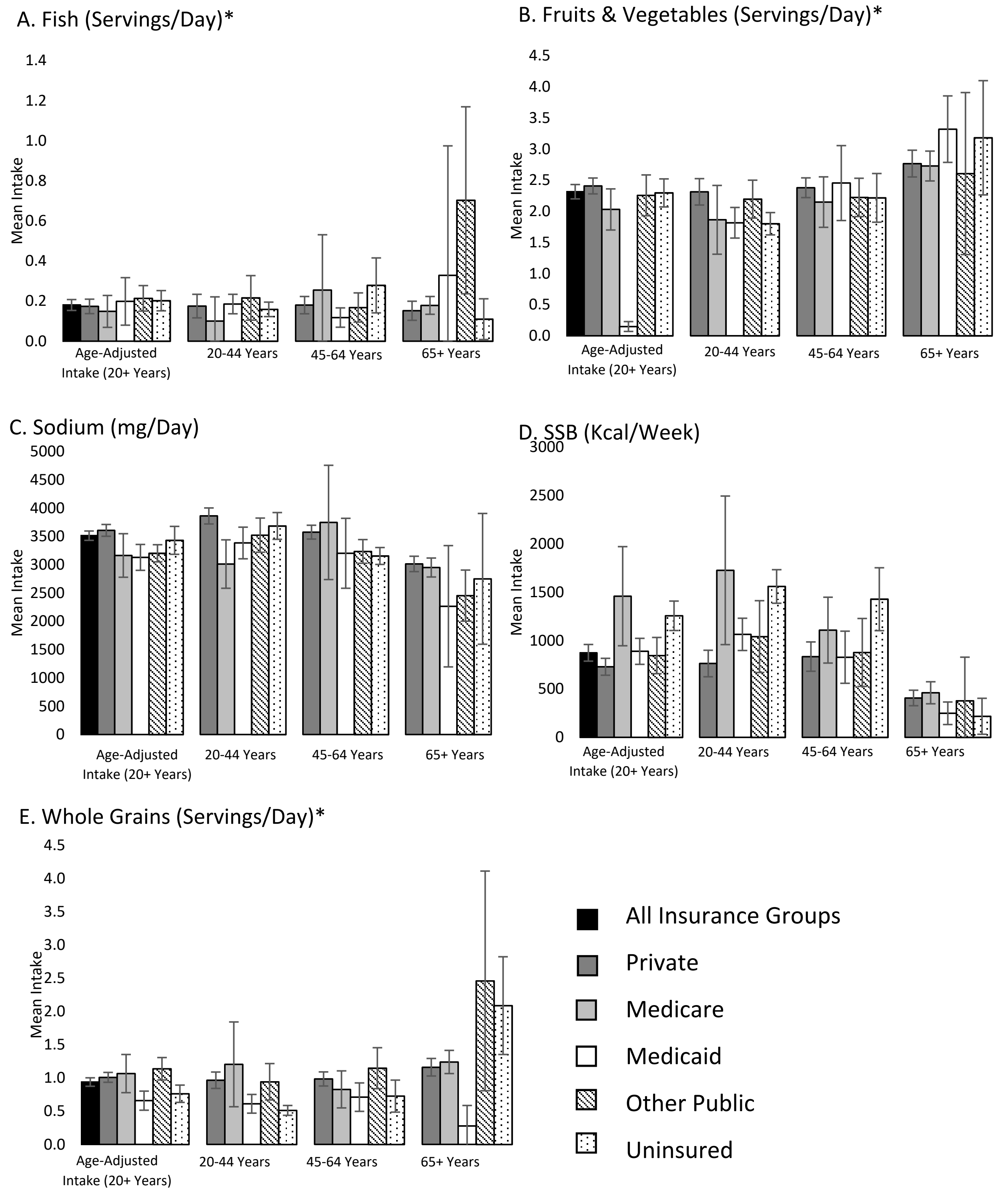


*Intakes standardized to a 2,000 kcal/day diet; Error bars represent 95% Confidence Intervals
